# Carboplatin Induced Fatal Autoimmune Hemolytic Anemia: First Reported Case

**DOI:** 10.4021/wjon234w

**Published:** 2010-08-29

**Authors:** Sunil Dacha, Anil K Reddivari, Shadi Latta, Manjari Devidi, Nkemakolam Iroegbu

**Affiliations:** a Department of Internal Medicine, Saint Joseph Hospital, Chicago, IL, USA

**Keywords:** Autoimmune hemolysis, Carboplatin

## Abstract

Carboplatin is an alkylating anti-neoplastic drug used in various cancers especially ovarian cancer, germ cell tumors, endometrial cancer besides others. We present a case of acute autoimmune hemolytic anemia during Carboplatin infusion in a patient previously exposed to the drug, resulting in the death of the patient. Published reports of Carboplatin induced autoimmune hemolytic anemia suggest these are usually nonfatal and improve after discontinuation of the drug. Fatal autoimmune hemolysis from Carboplatin has not been reported to the best of our knowledge. A 77-year-old Caucasian lady with history of endometrial adenocarcinoma was receiving treatment with a combination of Carboplatin and Paclitaxel for recurrent adenocarcinoma presenting as a pelvic mass. She tolerated similar chemotherapy previously, except for mild side effects. During her fifth cycle of chemotherapy with Carboplatin, she suddenly collapsed in the infusion center. Despite aggressive treatment, she expired within seven hours. A direct Coomb’s test was found to be positive. Carboplatin dependent antibody was also detected. She was felt to have had a Carboplatin-induced fatal hemolytic anemia. Acute autoimmune hemolytic anemia with Carboplatin is rare but could be a devastating complication. A sudden drop in hemoglobin during Carboplatin infusion should alert clinicians of this extremely fatal possibility.

## Introduction

Carboplatin is platinum based alkylating chemotherapeutic agent effective in the treatment of ovarian cancer, germ cell tumors, and endometrial cancer besides others. It is known to cause dose related myelosupression presenting as anemia, leucopenia or thrombocytopenia but immune hemolysis is rarely reported. An exhaustive search of PUBMED did not reveal any cases of fatal hemolytic anemia caused by Carboplatin, with both reported cases of hemolysis from Carboplatin being of a nonfatal nature [1, 2].

Here, we present to our knowledge, the first fatal case of acute autoimmune hemolytic anemia during Carboplatin infusion in a patient previously exposed to the drug revealing the possibility of fatal immune hemolysis with repeated Carboplatin therapy.

## Case Report

A 77-year-old woman was diagnosed with T1N0 Mx papillary serous endometrial adenocarcinoma. She underwent total abdominal hysterectomy, bilateral salphingo-oophorectomy, and lymph node dissection in 2000. In 2006, following an abnormal Pap smear, a biopsy of the vaginal cuff revealed metastases to this region. Positron emission tomography (PET) scan showed recurrence of the pelvic tumor, with lymph node metastases, and obstructive uropathy. Bilateral ureteral stenting was performed and the patient was treated with Carboplatin and Paclitaxel infusions from November 2006 to April 2007. The treatment was tolerated well except for the development of chronic anemia for which she received three units of packed red cell transfusion. In September 2007, a follow-up PET scan showed a recurrence of the pelvic tumor for which the patient was restarted on cyclical chemotherapy with weekly infusions of Carboplatin and Paclitaxel. During the fifth cycle of Carboplatin infusion, she became unresponsive and hypotensive. Carboplatin infusion was stopped and the patient was transferred to the emergency department (ED) at the same institution. In the ED she was unresponsive, with a blood pressure of 60/30 mmHg, heart rate of 110 beats per minute, respiratory rate of 26 breaths per minute, and oxygen saturation of 85% on 50% oxygen by ventimask. On physical examination she looked pale, with cool and clammy extremities. Her lungs were clear and the rest of the physical examination was normal. Laboratory tests showed: a hemoglobin of 3.8 g/dl (Her hemoglobin just prior to the Carboplatin infusion was 10.4 g/dl), arterial blood gases (ABG) were pH of 6.97, PCO2 of 14.4 and PaO2 of 271.7 mm Hg. Sodium was 130 mmol/lit (normal value 135 - 145 mmol/lit), with potassium of 4.6 mmol/lit (normal value 3.5 - 5.00 mmol/lit), chloride of 95 mmol/lit (normal value 97 - 108 mmol/lit), bicarbonate of 11 mmol/lit (normal value 22 - 29 mmol/lit), blood urea nitrogen of 32 mg/dl (Normal value 5-20 mg/dl), creatinine of 1.57 mg/dl (normal value 0.5 - 1.0 mg/dl), negative troponins and a slightly elevated creatinine kinase levels. Head CAT scan was normal. Peripheral blood smear showed clumped red blood cells (RBC’s). Serologic studies were sent for direct and indirect coombs test. The patient was intubated, and received three units of packed red blood cells and was resuscitated with intravenous normal saline, intravenous bicarbonate solution, steroids, and vasopressors. Despite aggressive treatment, she expired in seven hours. Later on, her serum was found to be positive for direct Coomb’s test. Carboplatin-dependent antibody was also detected. Direct anti-globulin (Coomb’s) test (DAT) was positive and showed the presence of anti-IgG and anti-C3 on the patient’s RBC’s. Elute prepared from patient’s RBC’s reacted when tested against red blood cells in the presence of Carboplatin and did not react with other cells, supporting an “immune complex-mediated destruction of RBC’s. The patient’s serum reacted with untreated RBC’s in the presence of Carboplatin, with titers at room temperatures and 37o Celsius of 2048. Titers for the antiglobulin test was 1024, supporting the presence of Carboplatin-dependent antibody and the diagnosis of Carboplatin-induced fatal hemolytic anemia.

## Discussion

Drug induced autoimmune hemolytic anemia (AIHA) is a well known complication, with the first case reported back in 1954 with fuadin, an anti-schistosomal medication. Since then, over 100 drugs have been reported to cause AIHA of which the common agents are second and third generation cephalosporins, the penicillins and derivatives, diclofenac, beta-lactamase inhibitors, fludarabine, cisplatin, and quinine, etc [3]. The incidence of drug induced AIHA has been estimated up to 1: 1,000,000 [4].

Drug and /or drug metabolites interact with RBC membranes causing composite immunogenic epitopes that can elicit antibody production. Autoantibody induced by the drug may be either drug dependent or drug-independent. The antibody can react to drug-treated RBCs (drug-adsorption mechanism), untreated RBC’s in the presence of drug (immune complex mechanism) and untreated RBC’s in the absence of the drug (true autoantibody mechanism) [3].

Carboplatin is an alkylating anti-neoplastic drug used in various cancers, especially ovarian cancer, germ cell tumors and endometrial cancer among others. It is known to cause a dose-related myelosuppression resulting in anemia and thrombocytopenia, but immune hemolysis is rarely reported in the literature [1, 2]. Cisplatin and oxaloplatin are chemically related chemotherapeutic agents also reported to cause immune hemolysis [5-8]. The role of Carboplatin as a cause of immune hemolysis in our patient was evident from the sudden drop in hemoglobin during its infusion with later confirmation by the presence of Carboplatin-dependent antibodies, making this the first of its kind and revealing the possibility of death from severe immune hemolysis with repeated Carboplatin therapy. Anemia during Carboplatin therapy has been frequently reported and is usually mild, increasing with the number of chemotherapy cycles. The effect on RBC production may be cumulative and delayed. The mechanism of sensitization to platinum salts remains unclear. There have been a few reports in the literature implicating Cisplatin in the development of DAT and drug induced hemolysis [9]. This case shows that autoimmune hemolysis can occur after repeated cycles of Carboplatin therapy. Patients who experience expected symptoms or signs during Carboplatin infusion should probably have the drug stopped and additional laboratory data obtained. A sudden decrease in hemoglobin during Carboplatin therapy should raise suspicion of immune hemolysis. Management depends on the severity and the response to treatment. Besides stopping the offending drug, options include IV steroids, IVIG, splenectomy, plasma exchange, immunosuppressant therapy and monoclonal antibody treatment as is done with other types of autoimmune hemolytic anemia.

## Abbreviations

RBC: Red blood cell
ED: Emergency department
PET: Positron emission tomography
IAT: Indirect antiglobulin test
